# Differential variability and correlation of gene expression identifies key genes involved in neuronal differentiation

**DOI:** 10.1186/s12918-015-0231-6

**Published:** 2015-11-19

**Authors:** Tatsuya Ando, Ryuji Kato, Hiroyuki Honda

**Affiliations:** Department of Biotechnology, Graduate School of Engineering, Nagoya University, Nagoya, Aichi Japan; Department of Basic Medicinal Sciences, Graduate School of Pharmaceutical Sciences, Nagoya University, Nagoya, Aichi Japan

**Keywords:** Network biology, Systems biology, Dynamics, Stem cell, Differentiation

## Abstract

**Background:**

Understanding the dynamics of stem cell differentiation processes at the molecular level is a central challenge in developmental biology and regenerative medicine. Although the dynamic behaviors of differentiation regulators have been partially characterized, the architecture regulating the underlying molecular systems remains unclear.

**Result:**

System-level analysis of transcriptional data was performed to characterize the dynamics of molecular networks in neural differentiation of stem cells. Expression of a network module of genes tightly co-expressed in mouse embryonic stem (ES) cells fluctuated greatly among cell populations before differentiation, but became stable following neural differentiation. During the neural differentiation process, genes exhibiting both differential variance and differential correlation between undifferentiated and differentiating states were related to developmental functions such as body axis development, neuronal movement, and transcriptional regulation. Furthermore, these genes were genetically associated with neuronal differentiation, providing support for the idea they are not only differentiation markers but could also play important roles in neural differentiation. Comparisons with transcriptional data from human induced pluripotent stem (iPS) cells revealed that the system of genes dynamically regulated during neural differentiation is conserved between mouse and human.

**Conclusions:**

The results of this study provide a systematic analytical framework for identifying key genes involved in neural differentiation by detecting their dynamical behaviors, as well as a basis for understanding the dynamic molecular mechanisms underlying the processes of neural differentiation.

**Electronic supplementary material:**

The online version of this article (doi:10.1186/s12918-015-0231-6) contains supplementary material, which is available to authorized users.

## Background

**Fig. 1:**
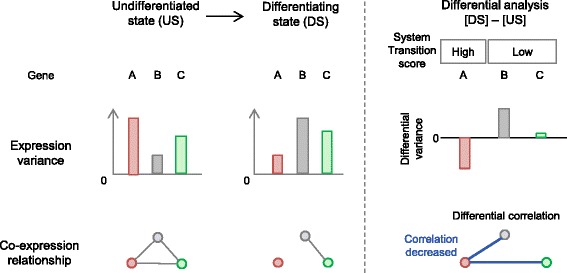
Methodological overview of system transition scoring based on network dynamics. Two indexes were used for system transition scoring: gene expression variance within replicates in each cellular state, and the co-expression relationship between genes in each cellular state. After calculating these indexes for each state, a differential analysis was performed to compare the indexes of the undifferentiated and differentiated states. A conceptual gene expression variance and co-expression network of three genes (genes A, B, and C) is shown. In the left panel, gene A (in red) exhibits the largest change in expression variance within replicates at the undifferentiated state (US), however the variance greatly decreases following the shift to the differentiating state (DS). This change in variance is defined as differential variance. Co-expression relationship (grey lines connecting three genes) is defined as the correlation between genes within replicates; therefore, the relationship between gene A and gene B/C diminishes in the DS. Such a correlation difference between states is defined as differential correlation (blue line indicates “decrease” of correlation” in the right panel). When both the differential variance and differential correlation are large, the system transition score is high. Gene B (in grey) and gene C (in green) are member genes that co-express with gene A in the undifferentiated state. Although gene B exhibits differential variance, the differential correlation of gene B is smaller than that of gene A. The differential variance of gene C is much smaller than that of gene A. The system transition scores of genes B and C are lower than that of gene A

Cell differentiation is a complex process requiring precise dynamic regulation of cellular components. The spatio-temporal heterogeneity of ES cells and iPS cells makes it hard to determine the molecular mechanisms of cell differentiation and establish efficient differentiation protocols [1, 2]. From the standpoint of dynamic systems theory, differentiation processes, like societies and ecological or biological networks, are systems that shifts abruptly from one state to another, often in response to external stimulation; such shifts are referred to as “critical transitions” [3–5]. ES and iPS cells are in a balanced stable state that can shift to multiple other states representing differentiated cell types [5, 6]. Although it may exhibit little change beforehand, a system close to a critical transition usually shows signs of fragility. For example, high variance and correlation of system components are empirical indicators of upcoming transitions [4]. Thus, the mRNA and protein components of a gene regulatory network may exhibit highly variable expression and correlated expression patterns prior to ES and iPS cell differentiation. Such variance is thought to be related to spatio-temporal fluctuation (“noise”) in gene expression [7]. Controlled temporal fluctuation or oscillation is required for maintenance of stem cell self-renewal [8]. Some gene sets related to self-renewal not only oscillate, but are also co-expressed during neural differentiation [9]. The co-expression network provides a comprehensive picture of the correlation relationships between gene products and reveals the functional organization of the transcriptome [10, 11]. The structure of the transcriptional regulatory network may be altered during ES cell differentiation. Fluctuations in the levels of important differentiation regulators may affect network structure, thereby controlling cell fate decisions and population heterogeneity [12]. Recently developed systematic approaches can identify such changes in network structure [13]. These approaches identify regulators or marker genes of disease pathophysiology by comparing gene network structures and variances of the genes in the network between healthy and disease progression states [14–20]. Application of these methods to ES and iPS cell may reveal alterations in network structures and variance in gene expression levels during ES cell differentiation process, ultimately leading to identification of important regulators of differentiation. In support of this idea, several known regulators exhibit co-regulated fluctuations during differentiation [9]. Understanding of these gene regulatory networks could help dissect the complex molecular mechanisms underlying stem cell biology. Although pioneering work has revealed the behaviors of dynamic gene fluctuations, especially in developmental biology, genome-wide discovery of genes exhibiting dynamic fluctuation during differentiation has not been comprehensively performed. In this study, we developed an analytical framework for investigating the dynamics of transcriptional networks and applied it to the differentiation processes of ES and iPS cells. Examination of the gene expression profile during mouse neural differentiation revealed that the variability of a group of genes that were co-expressed in the undifferentiated state decreased after neural differentiation. We then ranked the individual genes using an integrative scoring method (Fig. 1) that simultaneously assessed the changes in gene expression variances and co-expression relationships between the undifferentiated and differentiated states. This analysis identified 671 highly ranked genes, including Hes1, previously shown to oscillate prior to neural differentiation. The common biological functions among these genes are related to neural differentiation, and act downstream of pluripotency-related transcription factors. This group was also enriched in genes that cause phenotypic alternations of developmental processes in KO/Tg mice. We demonstrated that these genes significantly overlapped with the set of genes that exhibited differential variance and correlation during neural differentiation of human iPS cells. This study suggests that analysis of network dynamics can be used to identify genes important for the differentiation process, as well as yield insights into dynamic molecular mechanisms in both mice and humans.

## Results

### Network dynamics detection in the differentiation process

**Fig. 2:**
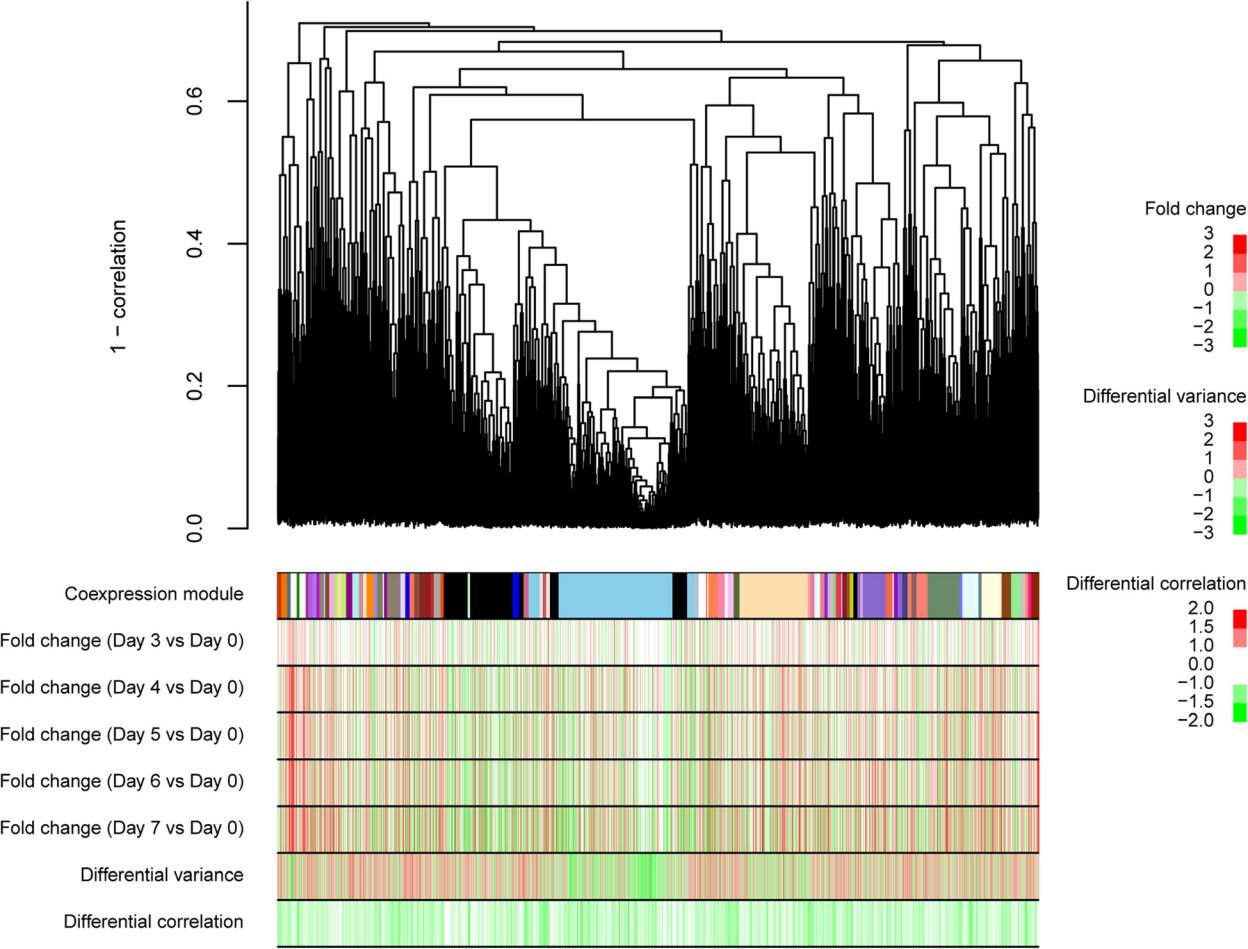
Co-expression modules identified in the undifferentiated state (day 0) in mouse ES cells. Dendrogram shows modules of co-expressed genes identified by hierarchical clustering of gene expression profiles on day 0. Individual colors represent single modules. The heatmap indicates fold change, differential variance, and differential correlation between day 3/4/5/6/7 and day 0. Differential variance indicates the absolute value of the difference between SD after day 0 and SD on day 0. Differential correlation indicates the absolute value of the difference between PCC after day 0 and PCC on day 0

We analyzed microarray data collected at six time points (days 0, 3, 4, 5, 6, and 7) from mouse ES cells undergoing neural differentiation; each time point was measured in eight replicates (E-TABM-1108) [21]. Two indicators were calculated to identify genes whose expression patterns predicted the transition from the undifferentiated state to the neural lineage (Fig. 1). One indicator was differential variance, representing the difference in gene expression variance between day 0 and each time point after day. The other was differential correlation, representing the difference between the average value of correlations within co-expressed gene sets at day 0 and those on subsequent days. These co-expressed gene sets, so-called ‘modules’, were defined at day 0 using hierarchal clustering. A system transition score was assigned to each gene by combining the differential variance and differential correlation (Fig. 1 and Methods). Hierarchical clustering analysis of gene expression data from ES cells at day 0 revealed 76 modules (Fig. 2). Comparisons of expression variances between day 0 and subsequent days identified 315 differentially variable genes (Additional file 1: Table S1). Genes for which the variances decreased after day 0 were the most significantly enriched in the skyblue module (colored sky blue in Fig. 2), which contained the most highly correlated genes at day 0 (*p* = 3.61e-50). This result indicates that genes specifically expressed in ES cells in the undifferentiated state were more variable than those expressed in differentiating cells, even though it is likely that multiple types of cells are present in the population during neural differentiation. On the other hand, the genes that were differentially expressed between day 0 and subsequent days were not enriched in the skyblue module (Fig. 2) as much as genes with differential variance (*p* > 0.999, Additional file 2: Table S2). As noted above, high differential variance and correlation are observed in fragile systems before a critical transition [4], and these features may represent an early warning signal of imminent differentiation.

### Genes with differential variance and correlation are involved in neural differentiation

**Fig. 3:**
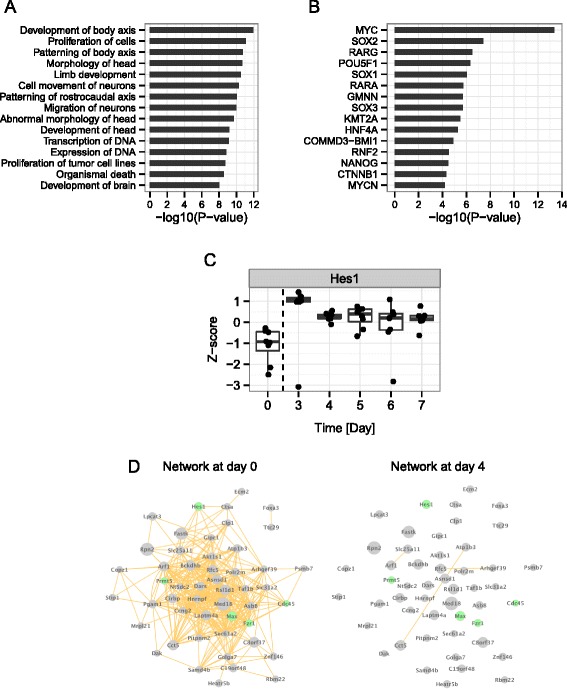
Functional analysis of DVC genes. **a** Functional categories enriched in the DVC genes during neural differentiation. **b** Upstream regulator analysis revealed transcriptional factors that could regulate the DVC genes. **c** Expression profile of a representative DVC gene, HES1. **d** Co-expression network of the module exhibiting the highest differential correlation and variance. Links between nodes represent strong correlation relationships (correlation coefficient ≥ 0.95). Green nodes indicate genes associated with differentiation-related phenotype in knockout or transgenic mice. Node size indicates system transition score

To identify fluctuating genes that contribute to dynamic changes in the transcriptional network, we ranked all genes based on their system transition scores. We referred to the highly ranked genes as DVC (**d**ifferential **v**ariance and **c**orrelation) genes. We detected 671 such genes (Additional file 3: Table S3). One of the top-ranked DVC genes, Hes1, exhibited high variance at day 0 (Fig. 3c). Co-expression relationships with Hes1 were diminished at day 4 relative to day 0 (Fig. 3d). Functional analysis of the DVC genes revealed that genes involved in body axis development, neuron movement, and transcription were enriched among the DVC genes (Fig. 3a). There were 822 differentially expressed genes that satisfied (1) p < 0.05 by t-test, and (2) fold change > mean + 2SD (standard deviation), when their mean expression levels were tested between differentiating states (Days 3, 4, 5, 6, and 7) and Day 0. Compared to the functions enriched in the DVC genes, the enriched functions of the differentially expressed genes included cancer-related functions (malignant solid tumor, proliferation of cells, digestive tumor, cell death of tumor) (Additional file 4: Table S4). Moreover, the DVC and differentially expressed genes did not significantly overlap (*p* = 0.793), but the DVC genes did significantly overlap with the set of genes (Fig. 3b) regulated by the Yamanaka factors (Myc, Sox2, and Pou5f1(Oct4)) expressed at the highest levels in the undifferentiated state (Day 0) (Additional file 5: Figure S1). Hoxb3, Fgf4, and Pax6 are downstream of both Pou5f1 (Oct4) and Sox2. These results suggest that the DVC genes not only represent early warning signals for neural differentiation, but are also functionally involved in the differentiation process.

### Gene expression variance and correlation are altered at a neuroectodermal stage

**Fig. 4:**
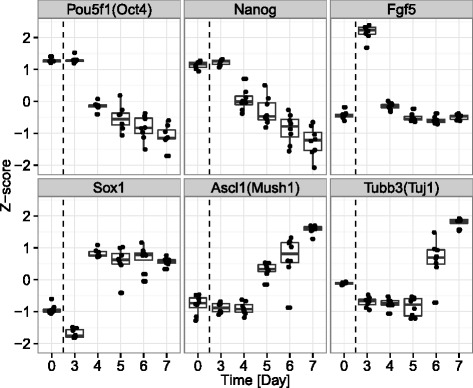
Expression profiles of neural differentiation markers in mouse ES cells. The Z-score indicates relative expression differences in each marker gene throughout the neural differentiation period (day 0 to day 7). Pou5f1 (Oct4) and Nanog are undifferentiated pluripotency markers. Fgf5 is a primitive ectoderm marker. Sox1 is an early neuroectodermal marker. Ascl1 (Mash1) and Tubb3 (Tuj1) are neural differentiation markers. Mouse ES cells pass through the epiblast-like stage at day 3 and convert to neurons at day 6

Next, we assessed the expression patterns of differentiation marker genes in order to understand when during the neuronal differentiation process their individual gene expression variance and co-expression relationship with associating genes changed. The cell populations initially expressed markers of the undifferentiated state, such as Pou5f1(Oct4) and Nanog, and began to express the primitive ectoderm marker Fgf5 on day 3 (Fig. 4). The early neuroectodermal marker Sox1 was expressed after day 4, and the neural markers Ascl1 (Mash1) and Tubb3 (Tuj1) were elevated after day 5 under neural differentiation conditions. Over half (55.7 %) of the DVC genes exhibited their largest changes in variability and correlation at Day 4. For instance, the expression variance of Hes1 decreased after day 4, when Sox1 was dramatically up-regulated. The co-expression networks of six time points are shown in Additional file 6: Figure S2. The figures show that the correlations between the genes in the skyblue module decreased the most at day 4. Because the early neuroectodermal marker Sox1 was highly expressed at Day 4, we believe that this time point represents a neuroectodermal stage. Based on these results, we infer that the transition to the neuroectodermal stage may involve an abrupt genetic system shift in differentiating cells.

### DVC genes are genetically associated with the differentiation process

**Fig. 5:**
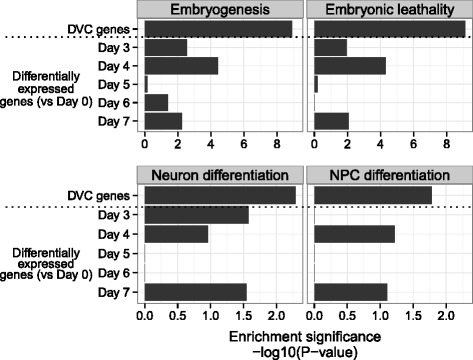
Genetic association of DVC genes with the differentiation process. Enrichment of gene sets associated with differentiation-related phenotypes in KO/Tg mice among the DVC and differentially expressed genes

We next performed enrichment analyses to determine whether the DVC genes play important roles in differentiation processes. To this end, we compared the DVC with the genes associated with differentiation-related phenotypes in knockout and transgenic mice. This analysis revealed that genes involved in embryogenesis, embryonic lethality, neural differentiation and neural progenitor cell differentiation were significantly more enriched among the DVC genes than among the differentially expressed genes (Fig. 5). Thus, the DVC genes could play important roles in neural differentiation. In other words, the genetic factors underlying neural differentiation could control differentiation by affecting the transcriptional network and its fluctuations, which are measured as dynamic changes in correlations and variances.

### DVC genes conserved in mouse ES and human iPS cells

**Fig. 6:**
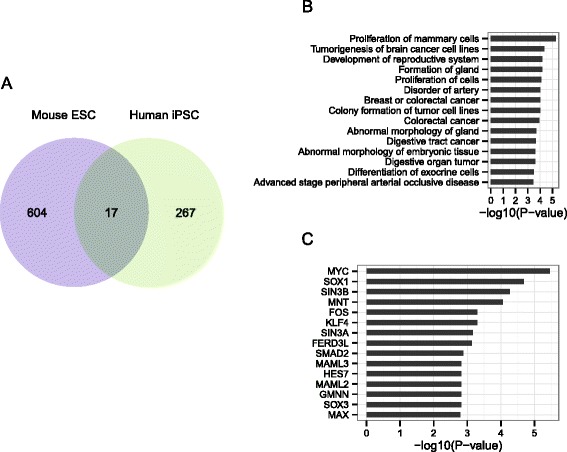
Common transcriptional regulators and functions of DVC genes in mouse ES and human iPS cells. **a** Venn diagram indicating the overlap between DVC genes from analyses of mouse ES and human iPS cells. DVC genes overlapped significantly between mouse ES and human iPS cells (*p* = 0.0204). **b** Functional categories enriched in overlapped DVC genes during neural development. **c** Upstream regulator analysis revealed transcriptional factors that could regulate the overlapping genes

Next, we assessed the conservation between humans and mice of differential variance and correlation during neural differentiation. To this end, the analysis used to detect network dynamics was applied to gene expression data collected at three differentiating states during neural differentiation of human iPS cells: iPS cells, neural precursor cells, and neurons [22]. We identified 284 DVC genes in neural differentiation of human iPS cells (Additional file 7: Table S5), which overlapped significantly with those in mice (*p* = 0.0204, Fig. 6a); for example, Hes1 and Ccng2 in the mouse co-expression network were also identified as DVC genes in neural differentiation of human iPS cells (Fig. 3d). Proliferation- and morphology-related genes were over-represented both in mouse and human (Fig. 6b). Furthermore, in both species, DVC genes were commonly regulated by MYC and SOX1 (Fig. 6c). For example, SOX1 is a transcriptional factor for HES1 [23], and the abrupt up-regulation of SOX1 may drive the dynamic changes of HES1 expression and co-expression relationships at day 4. Both genes play important roles in stem cell maintenance [24].

## Discussion

We carried out the first genome-wide analysis aimed at detecting dynamical changes in gene-expression variance and co-expression relationships during neural differentiation of mouse ES and human iPS cells. Our results demonstrate that genes that were highly correlated in ES cells exhibited significant changes in expression variance. Functional analysis revealed that genes exhibiting both differential variances and differential correlations may encode the regulators of neural differentiation. Although differentially expressed genes are normally used to identify genes that play important roles in differentiation [25–27], analysis of network dynamics allows us to identify potential key regulators that cannot be detected by differential expression analysis.

The DVC genes tended to be downstream of the Yamanaka factors (Fig. 3), and could therefore be related to self-renewal of stem cells and maintenance of pluripotency. Hoxb3, Fgf4, and Pax6 are downstream of both Pou5f1 (Oct4) and Sox2. Hoxb3 plays a role in maintaining self-renewal [28], and Fgf4 is involved in pluripotency [29]. On the other hand, Pax6 is a master regulator of neuronal differentiation [30]. Sox1, one of the common transcription factors upstream of the DCV genes in both the mouse and human studies (Fig. 6), promotes neurogenesis [23]. Hes1 and Pitx2 are downstream of Sox1. Hes1-null mice exhibit premature neurogenesis and neural tube defects during embryogenesis [31]. The Hes1 protein is a transcriptional repressor that inhibits differentiation of ES cells into the neural lineage and delays mesoderm and endoderm differentiation [32]. Pitx2 is important for mesodermal and neuroectodermal development *in vivo* [33]. These results suggest that the DVC genes include not only pluripotency genes but also the genes specifically related to neuronal differentiation.

SOX1 also acts upstream of DVC genes in human iPS cells. HES1, FRZB, and WLS are common DVC genes downstream of SOX1 in both humans and mice. Reduction in FRZB expression is required for neural progenitor proliferation and the acceleration of neuron development [34]. FRZB can bind extracellular WNT and inhibits Wnt signaling. WLS is important for Wnt-mediated neuronal development [35]. These genes might have regulatory links and be involved in a neuronal differentiation.

HES1 expression is dynamically regulated during neuronal differentiation. In particular, Hes1 expression oscillates in ES cells and neural progenitor cells, and is transiently down-regulated during the transition to epiblast stem cells (epiSCs) in neural and non-neural lineages [9, 32, 36]. The heterogeneity of Hes1 expression was greater in the ES cell population than in epiSCs. This heterogeneity may be due to oscillatory expression, observed as expression variances in ES cell populations. Our analysis was able to detect the change in expression variances during the transition from ES cells to the neural lineage.

Inactivation of HES1 in ES cells promotes rapid and homogeneous differentiation into neural progenitors [36]. CCNG2 was also identified as a DVC gene in both mouse ES and human iPS cells. Both Hes1 and Ccng2 prolong G1 phase to reduce cell proliferation [37, 38]. Smad2 is a transcriptional regulator of Hes1 and Ccng2, and the inhibition of Smad2 promotes immediate differentiation into functional neurons [39]. These findings suggest that modulation of the DVC genes might facilitate the development of experimental protocols for rapid and homogeneous differentiation of neurons. Because the heterogeneities of ES and iPS cells preclude the use of these materials as a stable supply of rapidly differentiated neurons, such an approach could contribute greatly to the understanding of brain functions and the development of regenerative medicine. These heterogeneities could arise in part from the dynamical behaviors of cellular components such as protein expression and localization. Recently, a growing number of attempts have been made to control dynamical patterns by targeted perturbations using chemical compounds and other interventions [40]. Perturbation of the genes identified by the methods in this study could contribute further to understanding of the molecular basis of stem cell differentiation. Moreover, dynamical regulations of the genes by such perturbations might control neural differentiation. Specific dynamical patterns are associated with various cellular responses such as apoptosis and immune response; therefore, the application of this method to other biological responses could identify important regulators of specific cellular responses.

However, it remains challenging to identify dynamic changes in molecular networks. The approach used in this study might not detect all the early warning signals of upcoming transitions. For example, genes previously shown to exhibit oscillatory expression in stem cells, such as Ascl1 and Dll1, were not identified in this study. One possible reason for this is that we measured mean values of gene expression in cell populations, potentially resulting in underestimation of gene expression variance among individual cells. A single-cell analysis of gene-expression profiles with a large number of replicates would help us to observe the transcriptional distribution of each gene across individual cells. Characterizing the transcriptional distribution in this manner could provide more accurate estimation of a gene expression variance between cellular states.

There are, of course, genetic and epigenetic differences between humans and mice. For example, the molecular machineries that maintain the stemness of ES and iPS cells are not completely identical [27, 41]. In addition, various human iPS cell lines come from different genetic backgrounds [22]. Despite such differences, a significant number of common DVC genes were identified during neural differentiation in mouse ES and human iPS cells. This finding supports the idea that the machineries responsible for dynamic changes in gene expression variances and correlations during neural differentiation are conserved between humans and mice.

## Conclusions

Our system-level analysis of the mouse ES and human iPS cell transcriptomes demonstrates the existence of a transitional state during neural differentiation that exhibits fluctuations in gene expression and transcriptional regulation. These dynamic transcriptional changes, identified by unbiased systematic detection of early warning signals for upcoming neural differentiation, were genetically related to developmental functions. Our analysis provides a systems biology framework that could be used to gain insights into the mechanisms underlying dynamic developmental processes in stem cells.

## Methods

### Transcriptional data

Transcriptional data of neural differentiation in mouse ES cells were obtained from the ArrayExpress database (E-TABM-1108) [21]. Transcriptional data of neural differentiation in human iPS cells were obtained from the Gene Expression Omnibus (GSE25542) [22]. The raw transcriptional data were log_2_ transformed and subjected to quantile normalization. Probes corresponding to genes that were expressed (presence call > 50 %) and exhibited variance (SD > 0) across replicates in each time point or differentiation stage were used in the analysis. Differential co-expression and variation analyses were performed based on 30,035 and 12,364 probes from mouse ES cell and human iPS cell data, respectively. The gene expression signals were standardized to the Z-score (average = 0, SD =1) for each gene across replicates of each time point or differentiation stage.

### Differential variance and co-expression analysis

The differential co-expression and variation analysis was conducted using the Bioconductor package in the R language [42]. To identify modules, hierarchical clustering was applied to the standardized expression values from mouse ES cells at day 0 or from human iPS cells. The hierarchical clustering was performed based on the Pearson correlation coefficient (PCC) and average linkage method. Modules were detected using a dynamic tree-cutting algorithm (hybrid mode, minimal module size of 100). In each module, the average PCC of each gene with other genes in the module was calculated. Differential correlation was calculated as the absolute value of the difference between PCC after day 0 and PCC at day 0 in the module as defined on day 0. The average SD of each gene among replicates in each time point or cellular state was calculated. The differential variance was calculated as the absolute value of the difference between SD after day 0 and SD at day 0. The system transition score, based on a previously described composite index, was used to rank the genes and identify those with high differential correlation and variance during neural differentiation [15]. This score was calculated using in the following formula:

$$ System\kern0.20em transition\kern0.20em score=\kern-0.20em  \max \left\{ \log \left(\frac{\frac{PCC\ast SD}{OPCC}\kern0.24em  at\kern0.24em  day\kern0.24em 0}{\frac{PCC\ast SD}{OPCC}\kern0.24em  at\kern0.24em  day\kern0.24em 3,\kern0.20em 4,\kern0.20em 5,\kern0.20em 6,\kern0.20em 7}\right)\kern-0.20em \right\} $$

where OPCC is the average PCC of each gene with the genes outside the module. Highly ranked genes were defined those whose scores were 2 SDs higher than the average score over all genes. Highly ranked genes in the same module displayed in network representation using Cytoscape 3.1.2 [43]. Correlation coefficients above 0.95 are shown as connections in the network visualization figure.

### Enrichment analysis

Functional enrichment and upstream regulator analysis was performed using Ingenuity Pathways Analysis (IPA®, Qiagen, http://www.ingenuity.com) software. Genes associated with differentiation-related phenotypes in knockout and transgenic mice were identified based on the Mouse Genome Informatics database [44]. Enrichment of genes associated with differentiation-related phenotypes among DVC genes was assessed by cumulative hypergeometric probability using the *phyper* function in R.

### Statistical analysis

Welch’s t-test was applied to transcriptional data to identify genes differentially expressed between day 0 and subsequent days during neural differentiation process. F-test was carried out to evaluate differential variances of genes between day 0 and subsequent days. P-values were adjusted by the Benjamini–Hochberg method [45]. Differentially expressed genes were defined as those whose fold changes were more than 2 SDs higher than the mean of all genes, with adjusted *p*-value < 0.05, as in the definition of DVC genes. To compare the mouse ES cell and human iPS cell data, mouse orthologs of the DVC genes from the human iPS cell study were identified based on information in the HUGO Comparison of Orthology Predictions database [46]. Overlap analysis of DVC genes in mice and human was performed by hypergeometric test, as in the enrichment analysis.

## Availability of supporting data

The data sets supporting the results of this article are available in the ArrayExpress database under accession no E-TABM-1108 and the Gene Expression Omnibus repository under accession no GSE25542.

## Additional files

Additional file 1: Table S1. 315 differentially variable genes from mouse ES cells. (XLSX 77 kb) Additional file 2: Table S2. 821 differentially expressed genes from mouse ES cells. (XLSX 287 kb) Additional file 3: Table S3. 671 DVC genes from mouse ES cells. (XLSX 86 kb) Additional file 4: Table S4. Functional enrichment of 821 differentially expressed genes from mouse ES cells. (XLSX 10 kb) Additional file 5: Figure S1. Gene expression profile of the Yamanaka factors during neuronal differentiation. (PDF 8 kb) Additional file 6: Figure S2. Co-expression networks at six time points. (PDF 1213 kb) Additional file 7: Table S5. 284 DVC genes from human iPS cells. (XLSX 62 kb)

## Abbreviations

ES: Embryonic stem
iPS: Induced pluripotent stem
DVC: Differential variance and correlation
SD: Standard deviation
epiSCs: Epiblast stem cells
PCC: Pearson correlation coefficient
