# Supplementary figures and images for: Differential variability and correlation of gene expression identifies key genes involved in neuronal differentiation
Source: BMC Syst Biol. 2015 Nov 19;9:82. doi: 10.1186/s12918-015-0231-6 (PMC4653947; doi:10.1186/s12918-015-0231-6)

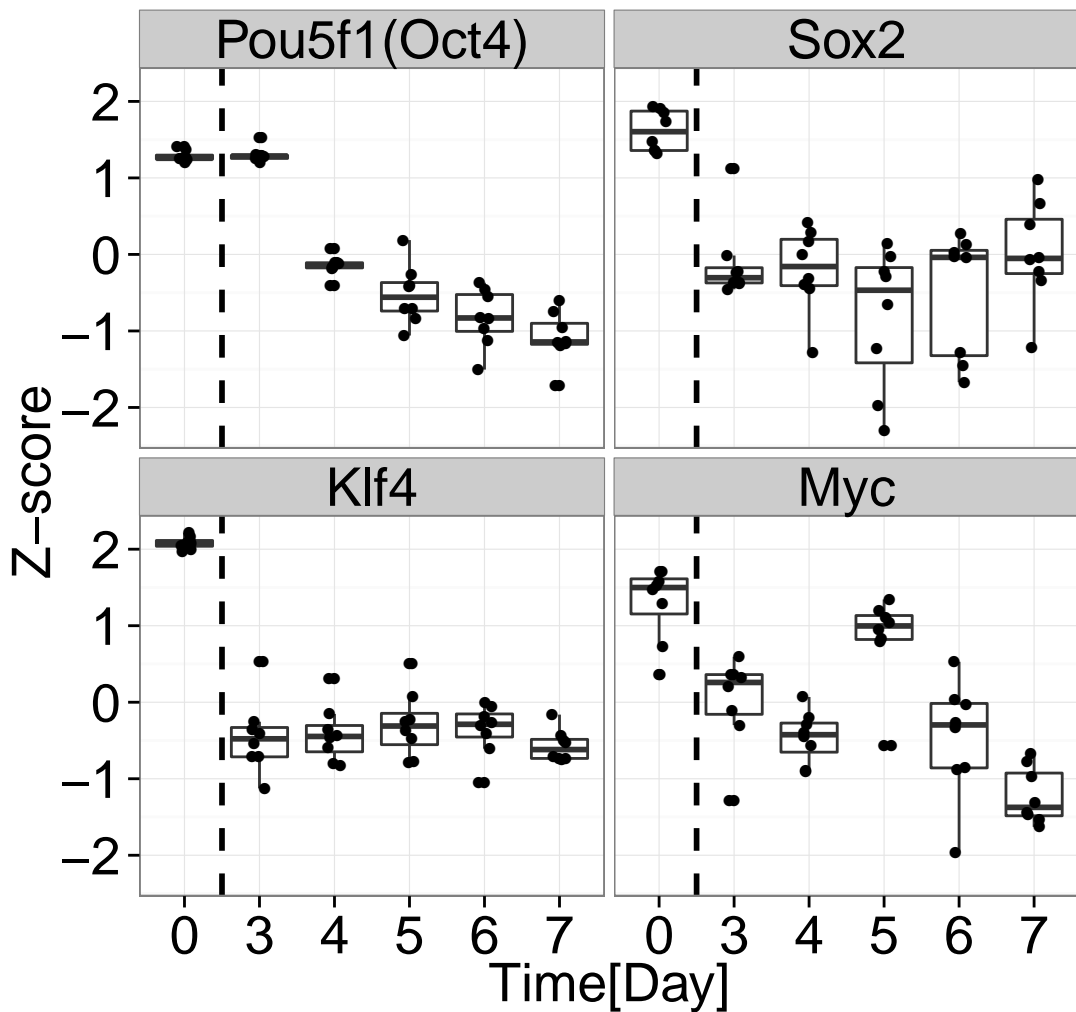

Supplement: Additional file 5: Figure S1. — Gene expression profile of the Yamanaka factors during neuronal differentiation. (PDF 8 kb) [file 12918_2015_231_MOESM5_ESM.pdf]

a

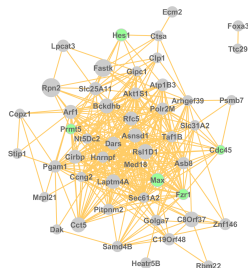

Network at day 0

b

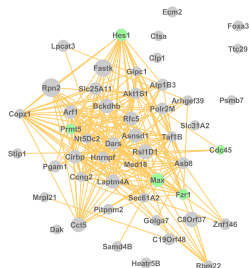

Network at day 3

c

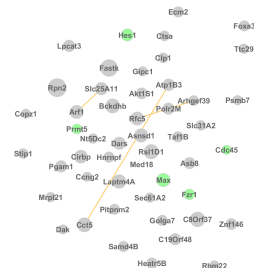

Network at day 4

d

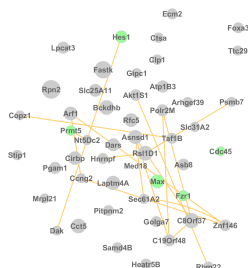

Network at day 5

e

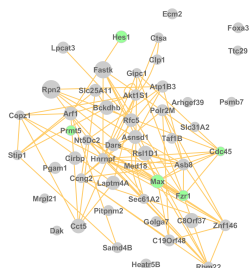

Network at day 6

f

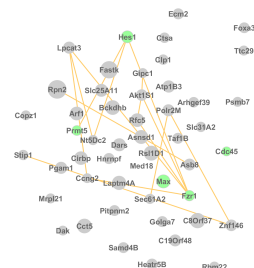

Network at day 7

Supplement: Additional file 6: Figure S2. — Co-expression networks at six time points. (PDF 1213 kb) [file 12918_2015_231_MOESM6_ESM.pdf]
